# Ephrin Receptors and Ephrin Ligands in Uveal Melanoma: A Big Data Analysis Using Web Resources

**DOI:** 10.3390/ijms27010442

**Published:** 2025-12-31

**Authors:** Georgios Mandrakis, Christina-Maria Flessa, Panoraia Keratsa, Apostolos Zaravinos, Stamatios Theocharis, Alexandros G. Sykaras

**Affiliations:** 1 First Department of Pathology, Medical School, National and Kapodistrian University of Athens, 75 Mikras Asias Street, Bld. 10, Goudi, 11527 Athens, Greece; giormandr@med.uoa.gr (G.M.); panorke@med.uoa.gr (P.K.); alexander.sykaras@gmail.com (A.G.S.); 2 Department of Biological Chemistry, Medical School, National and Kapodistrian University of Athens, 11527 Athens, Greece; cflessa@med.uoa.gr; 3 Department of Life Sciences, School of Sciences, European University Cyprus, 1516 Nicosia, Cyprus; a.zaravinos@euc.ac.cy; 4 Cancer Genetics, Genomics and Systems Biology Laboratory, Basic and Translational Cancer Research Center (BTCRC), 1516 Nicosia, Cyprus; 5 UCD School of Medicine, UCD Health Sciences Centre, University College Dublin, Belfield, Dublin 4, Ireland

**Keywords:** uveal melanoma, metastasis, EPH receptors, EFN ligands, molecular pathways, prognostic biomarkers

## Abstract

Uveal melanoma (UVM) is a rare cancer that represents the second most common melanoma (after the cutaneous) and the most common primary intraocular malignancy in adults. Despite recent advances in the understanding of UVM pathogenesis, its prognosis remains unchanged, with half of patients dying because of liver metastasis. Erythropoietin-producing human hepatocellular receptors (EPHs) constitute the largest known family of tyrosine receptors, and, along with their ligands, EFNs, regulate key physiological processes and are implicated in cancer pathogenesis. In this study, we used open-access web bioinformatics platforms to explore and analyze big datasets provided by The Cancer Genome Atlas (TCGA) UVM cohort of patients. We profiled the genomic alterations present in a subset of UVM patients, highlighting a likely pathogenic deep deletion of *EPHA7*. Survival analysis showed that overexpression levels of *EPHA4*, *EPHA5*, *EPHA8*, *EPHB2*, and *EFNB2* are significantly associated with poor overall survival. Additionally, high expression levels of *EPHA4*, *EPHA5*, *EPHA7*, *EPHA8*, *EPHB2*, *EFNA2*, and *EFNB2* correlate with reduced progression-free interval and disease-free survival. Finally, we identified the EPHs (*EPHA2*, *EPHA4*, *EPHA8*, and *EPHB4*) and EFNs (*EFNA1*, *EFNA3*, *EFNA4*, and *EFNB2*) that are significantly overexpressed in the aggressive epithelioid histological subtype and revealed that the majority of EPHs/EFNs are overexpressed in metastatic disease. In conclusion, our results highlight that a subset of EPHs and EFNs may be associated with worse clinical outcomes (*EPHA4*, *EPHA5*, *EPHA7*, *EPHA8*, *EPHB2*, *EFNA2*, and *EFNB2*), and an aggressive histological subtype (*EPHA2*, *EPHA4*, *EPHA8*, *EPHB4*, *EFNA1*, *EFNA3*, *EFNA4*, and *EFNB2*). The potential correlation of these genes with clinicopathological parameters of UVM need to be evaluated and validated with bioinformatic and experimental approaches in well-characterized cohorts of UVM patients.

## 1. Introduction

Uveal melanoma (UVM) is a malignant melanocytic neoplasm arising from the uveal tract, which comprises the iris and ciliary body anteriorly, and the choroid posteriorly. The choroid represents the site of origin for the vast majority of UVM cases [1]. Melanocytes are pigment-producing cells derived from the neural crest, which migrate to the developing eye between the 6th and 8th weeks of gestation and populate the uveal tract [2]. Although UVM is rare, it is the most common primary intraocular tumor in adults, accounting for approximately 5% of all melanomas and representing the second most frequent melanoma subtype, after cutaneous melanoma [3]. Most UVMs arise in the choroid and ciliary body, while iris melanomas are uncommon and differ in their molecular features, metastatic risk, and clinical management [4]. Despite effective local control through radiotherapy or surgery [5], metastatic spread, predominantly to the liver, remains the major cause of mortality, as almost half of patients with ciliary body or choroidal melanoma eventually develop metastatic disease [6,7]. Key clinicopathologic prognostic indicators include tumor size, ciliary body involvement, extraocular extension, and histological subtype, with spindle-cell tumors conferring more favorable prognoses than epithelioid or mixed-cell tumors [8,9]. These parameters constitute the staging criteria defined by the American Joint Committee on Cancer (AJCC), which classifies UVM into four stages (I–IV) [9].

Molecular characterization has revealed recurrent genetic alterations of prognostic importance in UVM. Gene expression profiling stratifies UVM into two groups, low and high metastatic potential [10,11]. Early driver mutations frequently affect the Gαq pathway genes *GNAQ* and *GNA11*, with some evidence suggesting that *GNA11* mutations may be associated with more aggressive disease [12,13]. Additional pathogenic mutations in *BAP1*, *SF3B1*, and *EIF1AX* define distinct, mutually exclusive prognostic subgroups, with *BAP1* loss linked to poor outcomes, and *SF3B1* or *EIF1AX* associated with more favorable prognoses [12]. Cytogenetic alterations, particularly monosomy 3, chromosome 8q amplification, and deletions in 1p and 16q, which represent major copy number alterations (CNAs), are strongly associated with metastatic progression [14,15]. Inactivating *BAP1* mutations, located on chromosome 3, are detected in the vast majority of metastatic tumors and correlate with characteristic morphological features, such as prominent lymphocytic infiltration, increased microvascular density, and nucleolar enlargement [16,17,18].

Comprehensive molecular profiling by The Cancer Genome Atlas (TCGA) further refined UVM classification into four molecular clusters with distinct genomic, epigenetic, and immunologic characteristics [19]. Cluster 1 typically includes disomy 3 tumors with *EIF1AX* mutations and low metastatic risk. Cluster 2 consists of disomy 3 tumors enriched in *SF3B1* mutations and associated with intermediate- or late-onset metastasis. Clusters 3 and 4 comprise high-risk tumors, characterized by monosomy 3, *BAP1* inactivation, 8q amplification, extensive chromosomal instability, and markedly increased metastatic potential [9]. This classification provides superior prognostic accuracy compared to AJCC staging [20] and is now used alongside clinical factors for risk stratification [21,22,23]. Despite advances in local therapies [24] and the development of systemic approaches, including chemotherapy, immunotherapy, and liver-targeted treatments [25,26,27], overall survival (OS) and disease-free survival (DFS) in UVM have not substantially improved over time [28,29]. Recent single-cell and integrative multi-omics studies have enhanced our understanding of UVM heterogeneity and have identified potential biomarkers and therapeutic targets [30,31,32,33,34,35], highlighting the ongoing need for improved prognostic biomarkers.

Erythropoietin-producing human hepatocellular receptors (EPHs) constitute the largest known family of plasma membrane receptor tyrosine kinases (RTKs). In humans, nine EPH-A receptors (EPHA1–8, EPHA10) and five EPH-B receptors (EPHB1–4, EPHB6) have been described. Their ligands, known as EFNs (EPH receptor–interacting proteins), are classified into two groups: EFN-A ligands, encoded by the *EFNA1*–*EFNA5* genes, and EFN-B ligands, encoded by the *EFNB1*–*EFNB3* genes. Typically, the A group of EFNs recognizes the matching A group of EPHs, while the B group of EFNs recognizes the EPHB receptors, although this stringent selection seems not to be decisive in some cases, such as EPHA4 and EPHB6 [36]. EFNs binding to EPHs usually require cell-to-cell interaction and result in bidirectional juxtracrine signal transduction, with forward signaling in the EPH-expressing cell, and reverse signaling in the EFN-expressing cell [37].

EPH/EFN signaling is implicated in myriad physiological processes that regulate tissue morphology and function [38]. The signaling pathways of the EPH/EFN system are linked to a variety of cellular and tissue functions, including remodeling and contraction of actin–myosin microfilaments, angiogenesis, synaptogenesis, and axon guidance in the nervous system, as well as cell proliferation, migration, adhesion, differentiation, and survival [39]. The critical role of the EPH/EFN system in cell proliferation, motility, and angiogenesis highlights the potential impacts of these signaling molecules in cancer. The EPH/EFN family has been extensively studied in many cancers and is considered as a source of potential cancer biomarkers with prognostic and therapeutic importance [40]. In cancer, overexpressed EPHs can activate signal transduction pathways (Ras/MAPK, Rho GTPase, and PI3K/Akt) without interacting with EFN ligands [36]. Additionally, both EPHs and EFNs have been reported to have dual roles in cancer development and progression. Specifically, distinct patterns of function have been discovered, as the *EPH/EFN* genes may act as oncogenes or tumor suppressors [41]. Thus, it appears that variable expression patterns of *EPH* and *EFN* genes can serve as molecular fingerprints for different tumor stages and histological subtypes [42].

Despite the plethora of studies that correlate *EPH/EFN* gene expression to clinicopathological parameters, very few studies have attempted to investigate the profile of EPH/EFN system in UVM [43,44]. The molecular and genetic characterization of UVM is required for patient stratification, as well as the identification of prognostic biomarkers and potential therapeutic targets [3,45]. However, the rarity of UVM poses challenges (insufficient recruitment of patients, resulting in small size of well-characterized UVM cohorts and scarcity of available experimental models), and the distinct features of UVM compared to cutaneous melanoma limit our progress [46]. The roles of *EPH/EFN* family genes in the pathogenesis of UVM, and their associations with the clinicopathological parameters of UVM patients, have not been explored. Our aim was to analyze the genomic and transcriptomic data generated by the TCGA-UVM project to identify members of EPH/EFN family with potentially important roles in UVM pathophysiology and clinical course. We employed bioinformatics open-access web platforms to uncover genes of the EPH network that may be associated with the pathogenesis and the clinical outcomes of UVM patients.

## 2. Results

### 2.1. EPH/EFN Pathogenic Variants (PVs) and Copy Number Alterations (CNAs) in UVM

**Figure 1:**
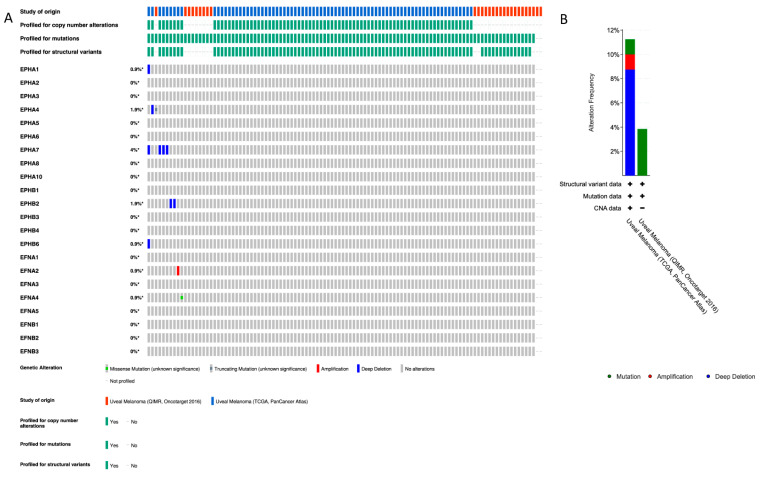
Profile of *EFN* and *EPH* gene pathogenic variants (PVs) and copy number alterations (CNAs) in UVM. (**A**) cBioPortal “Oncoprint” summary of PVs and CNAs in *EPH* and *EFN* genes in TCGA-UVM (n = 80) and QIMR-UVM (n = 28). Genomic alterations were infrequent in both UVM datasets. *EPHA7* (6q16.1) showed the highest CNA rate, with deep deletions in 4% of patients. Additional deep deletions were observed in *EPHA4* (2q36.1; 1.9%), *EPHB2* (1p36.12; 1.9%), and *EPHB6* (7q34; 0.9%). *EFNA2* (19p13.3) exhibited a single amplification event (0.9%). Point mutations were rare and included a truncating mutation (nonsense variant) in *EPHA4* (2q36.1; 0.9%) and a missense variant in *EFNA4* (1q21.3; 0.9%). Alterations are depicted with different colors (amplification, red; deletion, blue; mutation, green). Rows and columns depict genes and individual patients, respectively. Alteration frequency percentage is noted for each gene. (**B**) Alteration frequency percentage of *EPH* and *EFN* genes in TCGA-UVM (n = 80) and QIMR-UVM (n = 28). Alterations are depicted with different colors (amplification, red; deletion, blue; mutation, green). The asterisks indicate that not all the patients from the QIMR-UVM cohort were profiled for copy number alterations, mutations and structural variants.

We used cBioPortal database to profile the pathogenic variants (PVs) and CNAs of the EPH-/EFN-encoding genes that have been described in UVM patients (Figure 1A). The chromosomal locations of these genes are described in Table 1. Of the patients included in the TCGA-UVM study, 10% (8/80) have CNAs, with the vast majority of them being deep deletions of *EPH* genes (Figure 1B). The most common CNA present in UVM patients is the homozygous deletion of *EPHA7* (detected in 4/80 patients). *EPHA7* gene is located on the long arm of chromosome 6 (6q), loss of which is a recurrent chromosomal abnormality in UVM. *EPHA7* deep deletion is classified as a likely oncogenic/likely loss of function CNA by OncoKB and has also been identified in cutaneous melanomas, lymphomas, solid organ carcinomas (prostate, lung, and gastrointestinal tract adenocarcinomas), and neuroendocrine tumors (phaeochromocytomas and paragangliomas). The three patients with *EPHA7* homodeletion have UVM of mixed histological subtype and multiple cytogenetic abnormalities that result in multiple likely oncogenic deep deletions of other genes located at chromosome 6, chromosome 1, and chromosome 11. The fourth patient has spindle cell UVM, characterized by multiple deep deletions of genes located at chromosomes 6 and 7 (including *EPHA7*, *EPHA1*, and *EPHB6*), and likely oncogenic mutations in *GNAQ* and *SF3B1* genes. The other four patients have CNAs of unknown biological significance, according to OncoKB. Two patients with spindle cell UVM have deep deletions of *EPHB2*, and likely oncogenic mutations of *GNA11* gene. One patient has homodeletion of *EPHA4* has spindle cell UVM, characterized by additional CNAs and a pathogenic *GNAQ* point mutation. The only patient with a CNA other than deep deletion has amplification of *EFNA2*, a tumor of mixed histological subtype with numerous CNAs, and a pathogenic mutation of *GNAQ*.

Point mutations were detected in a lower percentage of patients compared to CNAs; 1/80 patients of the TCGA-UVM cohort and 1/28 patients of the QIMR, Oncotarget cohort have PVs of unknown biological significance. One spindle cell UVM patient harbors a nonsense PV in *EPHA4* gene (c.688C>T resulting in a premature stop codon at position 230 p.R230*), together with pathogenic mutations of *GNA11*, *SF3B1*, and *BAP1*. This EPHA4 mutation is a confirmed somatic PV that has been described in five cutaneous malignant melanoma patients [47], and in a patient with a serous endometrial carcinoma [48]. The other detected PV is a missense mutation (587G>A), causing the amino acid substitution R196H in EFNA4 protein of a spindle cell UVM patient with additional pathogenic PV of *GNA11*, *FBXW7*, *SF3B1*, and *SMARCA4* genes.

### 2.2. EPH/EFN Expression Correlates with Overall Survival, Disease-Free Survival, and Progression-Free Interval (OS, DFS, and PFI) in UVM Patients

Survival analysis performed by GEPIA2 (http://gepia2.cancer-pku.cn/#index, last accessed on 18 December 2025) [49] (for OS and DFS) and GEPIA3 (https://gepia3.bioinfoliu.com/, last accessed on 25 December 2025) [50] software (for OS and PFI) showed that upregulation of *EPH* expression is generally associated with worse outcome; however, this association is not significant for the majority of *EPHs*. Higher expression levels of *EPHA4* and *EPHA8* are significantly associated with decreased OS and DFS/PFI, whereas overexpression of *EPHA5* and *EPHB2* is associated with reduced OS and PFI. Overexpression of *EPHA7* correlates with decreased DFS/PFI, but not OS. Similarly to EPHs, overexpression of EFN genes is linked to worse prognosis. Higher expression of *EFNB2* is associated with worse OS and DFS/PFI, whereas higher expression of *EFNA2* is associated with decreased DFS/PFI. Single-gene survival analysis performed by GSCA (https://guolab.wchscu.cn/GSCA/, last accessed on 18 December 2025) also indicated that *EPHA4*, *EPHA5*, *EPHA8*, *EPHB2*, and *EFNB2* expression levels are associated with poor OS, while high expression levels of *EPHA4*, *EPHA5*, *EPHA7*, *EPHA8*, *EPHB2*, *EFNA2*, and *EFNB2* are associated with decreased PFI.

**Figure 2:**
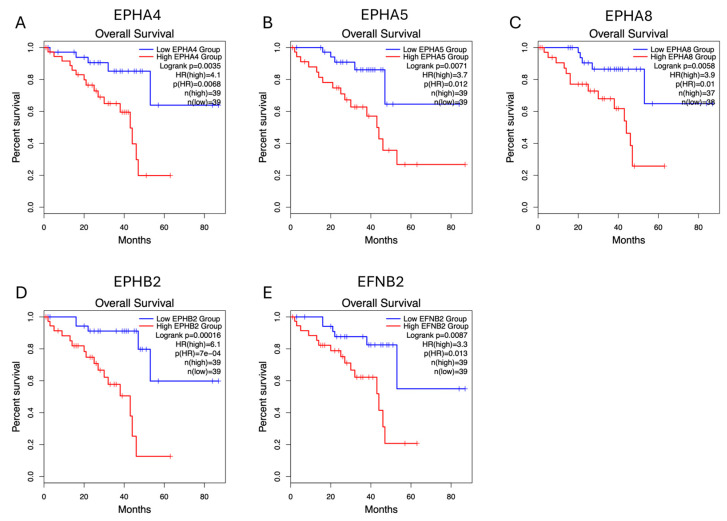
Overall survival (OS) impacts of *EPHA4*, *EPHA5*, *EPHA8*, *EPHB2*, and *EFNB2* expression levels in the TCGA-UVM cohort, analyzed by GEPIA2 online software (http://gepia2.cancer-pku.cn/, last accessed on 18 December 2025). Kaplan–Meier (KM) curves comparing OS of TCGA-UVM cohort patients with high mRNA expression (red) and low mRNA expression (blue) of *EPHA4* (**A**), *EPHA5* (**B**), *EPHA8* (**C**), *EPHB2* (**D**), and *EFNB2* (**E**). *p*-values and HRs were calculated by GEPIA2 using log-rank tests and Cox regression models. *p* < 0.05 was considered as statistically significant. TCGA-UVM, The Cancer Genome Atlas—Uveal Melanoma; GEPIA, Gene Expression Profiling Interactive Analysis; HR, Hazard Ratio.

**Figure 3:**
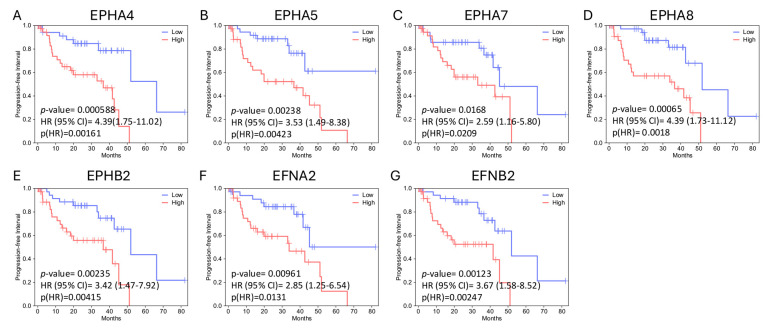
Progression-free interval (PFI) impacts of *EPHA4*, *EPHA5*, *EPHA7*, *EPHA8*, *EPHB2*, *EFNA2,* and *EFNB2* expression levels in the TCGA-UVM cohort, analyzed by GEPIA3 online software. Kaplan–Meier (KM) curves comparing PFI of TCGA-UVM cohort patients with high mRNA expression (red, n = 39) and low mRNA expression (blue, n = 39) of *EPHA4* (**A**), *EPHA5* (**B**), *EPHA7* (**C**), *EPHA8* (**D**), *EPHB2* (**E**), *EFNA2* (**F**), and *EFNB2* (**G**). *p*-values and HRs were calculated by GEPIA3 using log-rank tests and Cox regression models. Hazard Ratios (HRs) and 95% Confidence Intervals (CI) are shown on the graphs. *p* < 0.05 was considered as statistically significant. TCGA-UVM, The Cancer Genome Atlas—Uveal Melanoma; GEPIA, Gene Expression Profiling Interactive Analysis.

**Figure 4:**
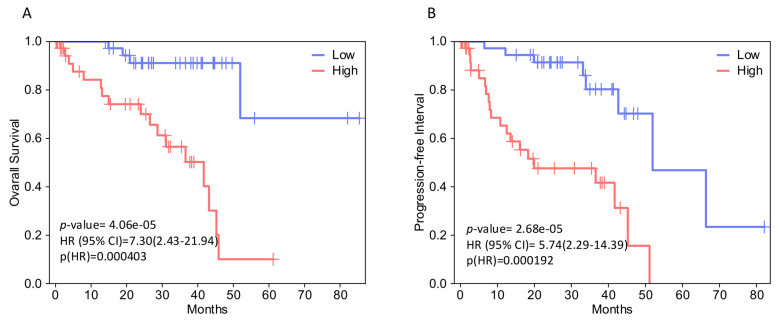
Survival analysis of selected EPH/EFN family member gene sets in TCGA-UVM patients, performed by GEPIA3 online software. (**A**) Kaplan–Meier (KM) curves comparing overall survival (OS) of TCGA-UVM cohort patients with high expression (red, n = 39) and low expression (blue, n = 39) of a gene set including *EPHA4*, *EPHA5*, *EPHA8*, *EPHB2*, and *EFNB2* (**B**) KM plots comparing progression-free interval (PFI) of TCGA-UVM cohort patients with high expression (red, n = 39) and low expression (blue, n = 39) of a gene set including *EPHA4*, *EPHA5*, *EPHA7*, *EPHA8*, *EPHB2*, *EFNA2*, and *EFNB2*. *p*-values were calculated by GEPIA3 using log-rank tests. *p* < 0.05 was considered as statistically significant. Hazard Ratios (HRs) and 95% Confidence Intervals (CIs) are shown on the graphs. TCGA-UVM, The Cancer Genome Atlas—Uveal Melanoma; GEPIA, Gene Expression Profiling Interactive Analysis.

In both analyses, *EPHA4*, *EPHA5*, *EPHA8*, *EPHB2*, and *EFNB2* (gene set 1) expression levels correlate with decreased OS. Figure 2 illustrates the Kaplan–Meier curves assessing the OS impact for each of these genes’ expression, generated by GEPIA2 software. The adjusted (for multiple-correction testing) *p*-values (q-values) were calculated by GEPIA3 [50] and are mentioned in Table S1. GEPIA2 and GSCA analyses agree that *EPHA4*, *EPHA5*, *EPHA7*, *EPHA8*, *EPHB2*, *EFNA2*, and *EFNB2* (gene set 2) are associated with reduced PFI (Figure 3). Next, we used the GEPIA3 tool to investigate if these gene sets’ expressions correlate with survival. This univariable gene set survival analysis revealed that there is a significant association between high expression of the genes constituting the sets and worse OS and PFI (Figure 4). We used TCGEx online tool (https://tcgex.iyte.edu.tr/, last accessed on 18 December 2025) [51] to perform a receiver operating characteristic (ROC) analysis to evaluate the potential prognostic significance of these gene sets for overall survival and metastasis-free survival. ROC analyses of gene set 1 and gene set 2 (except for *EPHA7*) demonstrated their potential prognostic utilities, with Area Under the Curve (AUC) values of 0.80 and 0.81, respectively (Figure S1). To investigate if EPHs/EFNs of interest have prognostic importance in other UVM cohorts, we used ShinyGeo [52] and performed a survival analysis on the dataset GSE84976, an Illumina expression array of 28 UVM patients [53]. This analysis is presented in Figure S2 and confirms the potential prognostic roles of *EPHA8*, *EPHB2*, and *EFNB2* in OS of UVM patients. Higher expression levels of *EPHA4* and *EPHA5* are also associated with worse OS, but not significantly.

### 2.3. EPH/EFN Expression in UVM Patients’ Subsets and UVM Molecular Clusters

**Figure 5:**
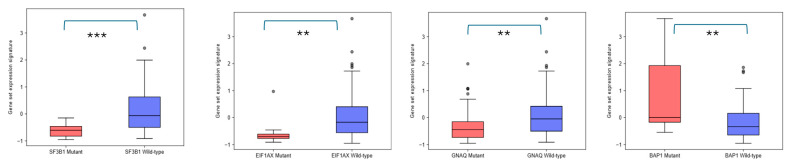
Differential expression of *EPHA4*, *EPHA5*, *EPHA8*, *EPHB2*, *EFNB2* gene set in TCGA-UVM patients harboring *SF3B1*, *EIF1A*, *GNAQ*, and *BAP1* mutations, performed by GEPIA3 online software. UVM patients with SF3B1 mutations (red, n = 18), EIF1AX mutations (red, n = 10), and GNAQ mutations (red, n = 39) have downregulated expression of this gene set compared to wild-type SF3B1 (blue, n = 61), EIF1AX (blue, n = 69), and GNAQ (blue, n = 40) patients. On the other hand, patients with BAP1 mutations (red, n = 13) overexpress these genes compared to BAP1 wild-type (blue, n = 66) UVM patients. *p* < 0.05 was considered as statistically significant. ** refers to *p* < 0.01; *** refers to 0.001.

We used GEPIA3 to analyze the expression pattern of the *EPH/EFN* gene set that is associated with OS. This analysis (gene set differential expression with hotspot mutation) revealed that the expression of the *EPHA4*, *EPHA5*, *EPHA8*, *EPHB2*, and *EFNB2* gene set is upregulated in UVM patients with *BAP1* mutations (associated with worse prognosis), and downregulated in UVM patients with *SF3B1*, *EIF1AX*, and *GNAQ* mutations (associated with better prognosis), compared to UVM patients who do not carry these pathogenic variants (Figure 5).

**Figure 6:**
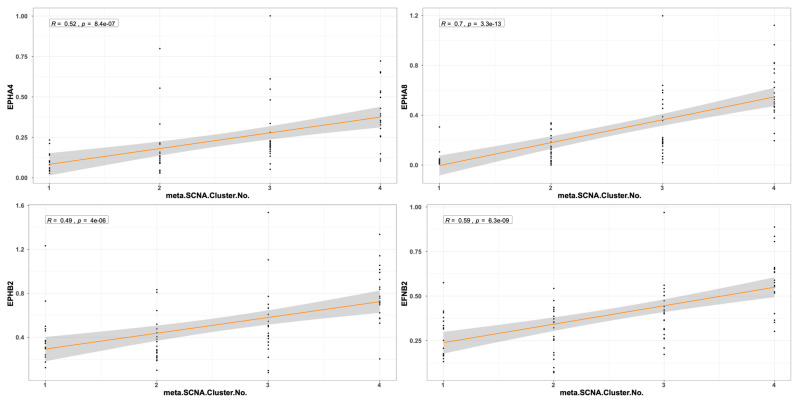
Scatterplot correlation analysis between UVM molecular cluster number (1, 2, 3, or 4) and levels of *EPHA4*, *EPHA8*, *EPHB2*, and *EFNB2*, performed by TCGEx software (https://tcgex.iyte.edu.tr/, last accessed on 18 December 2025). The analysis shows a significant positive correlation between cluster numbers and levels of EPH/EFN expression. R represents Spearman’s correlation coefficient; SCNA refers to somatic copy number alteration. The dark orange line represents the best fitting regression line and the grey area represents 95% confidence interval.

**Figure 7:**
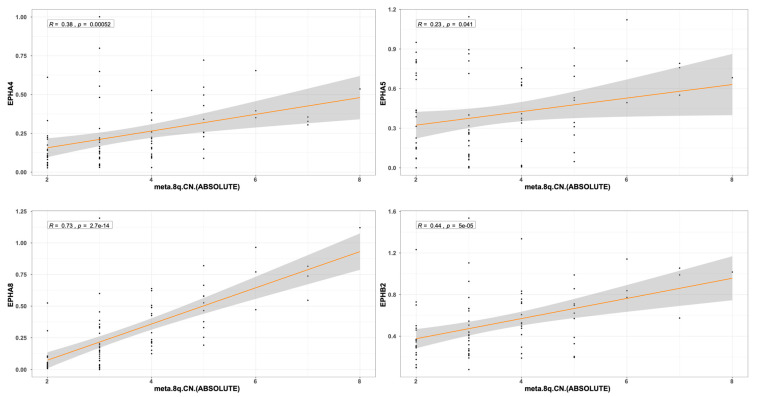
Scatterplot correlation analysis between 8q chromosome copy number in TCGA-UVM patients and levels of *EPHA4*, *EPHA5*, *EPHA8*, and *EPHB2*, performed by TCGEx software (https://tcgex.iyte.edu.tr/, last accessed on 18 December 2025). The analysis shows a significant positive correlation between cluster numbers and levels of *EPHA4*, *EPHA5*, *EPHA8*, and *EPHB2* expression. R represents Spearman’s correlation coefficient.The dark orange line represents the best fitting regression line and the grey area represents 95% confidence interval.

The expression pattern of *EPH/EFN* genes in *SF3B1*-, *EIF1AX*-, and *GNAQ*-mutated UVM prompted us to examine if their expression levels differ between the molecular clusters of UVM. We used TCGEx online tool [51] to perform a scatterplot correlation analysis to investigate if the selected *EPH/EFN* genes are upregulated in SCNA (somatic copy number alteration) molecular clusters 3 and 4, associated with *BAP1* mutations and worse prognosis. The correlation analysis revealed that there is a significantly positive correlation between cluster number and expression of *EPHA4*, *EPHA5*, *EPHA8*, *EPHB2*, and *EFNB2* (Figure 6). These results are in agreement with the analysis presented in Figure 5 and illustrate that these genes are downregulated in *SF3B1*-, *EIF1AX*-, and *GNAQ*-mutated UVM (clusters 1 and 2) and overexpressed in *BAP1*-mutated UVM (clusters 3 and 4). SCNA clustering is dependent on chromosomal aberrations, like 8q gain, a characteristic of cluster 3 and 4 UVM that is associated with worse prognosis. TCGEx scatterplot correlation analysis showed that 8q gain is significantly associated with overexpression levels of *EPHA4*, *EPHA5*, *EPHA8*, and *EPHB2* (Figure 7).

### 2.4. EPH/EFN Expression in Different Histological Subtypes of UVM

**Figure 8:**
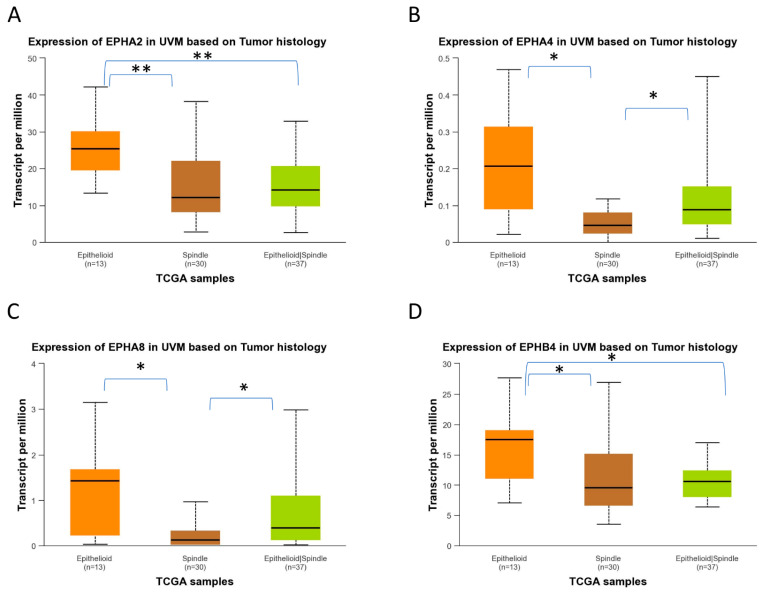
Differential gene expression levels of *EPHA2*, *EPHA4*, *EPHA8,* and *EPHB4* in epithelioid and spindle histological subtypes of TCGA-UVM samples, analyzed by UALCAN online software. (https://ualcan.path.uab.edu/, accessed on 18 December 2025) Boxplots depicting the mRNA expression levels of *EPHA2* (**A**), *EPHA4* (**B**), *EPHA8* (**C**), and *EPHB4* (**D**) in epithelioid (n = 13), spindle (n = 30), and mixed epithelioid/spindle TCGA-UVM samples (n = 37). Statistical analysis was performed by UALCAN with Welch’s (unequal variances) *t*-test comparison of pairs of samples. Significance is depicted as * *p* < 0.05, ** *p* < 0.01. UALCAN, The University of ALabama at Birmingham CANcer data analysis portal.

**Figure 9:**
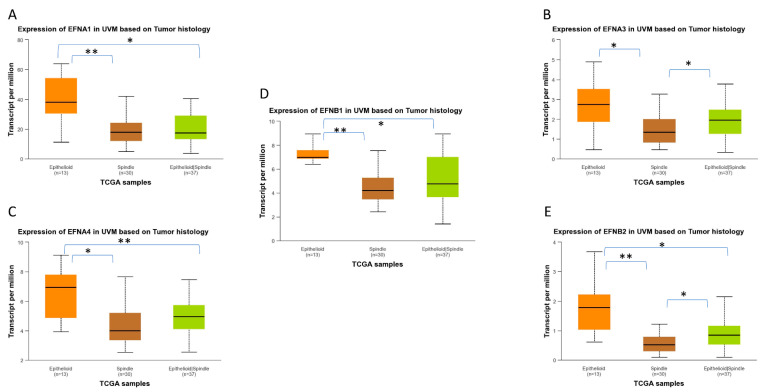
Differential gene expression levels of *EFNA1*, *EFNA3*, *EFNA4*, *EFNB1,* and *EFNB2* in epithelioid and spindle histological subtypes of TCGA-UVM samples, analyzed by UALCAN online software. The mRNA expression levels of *EFNA1* (**A**), *EFNA3* (**B**), *EFNA4* (**C**), *EFNB1* (**D**), and *EFNB2* (**E**) in epithelioid (n = 13), spindle (n = 30), and mixed epithelioid/spindle TCGA-UVM samples (n = 37), depicted as boxplots generated by UALCAN. Statistical analysis was performed by UALCAN with Welch’s (unequal variances) *t*-test comparison of pairs of samples. Significance is depicted as * *p* < 0.05, ** *p* < 0.01. UALCAN, The University of ALabama at Birmingham CANcer data analysis portal.

UALCAN-generated analysis of gene expression in different histological subtypes of UVM showed that *EPHA2*, *EPHA4*, *EPHA8*, and *EPHB4* mRNA expression levels are significantly upregulated in epithelioid UVM in comparison to spindle UVM (Figure 8). *EPHA5* expression is also associated with the epithelioid histology, but the adjusted *p*-value does not reach significance. The mixed histologic (epithelioid/spindle) subtype demonstrated significantly higher *EPHA4* and *EPHA8* expression levels in comparison with the spindle type (Figure 8B,C), and significantly lower expression levels of *EPHA2* and *EPHB2* compared to the epithelioid type (Figure 8A,D). Additionally, higher expression levels of *EFNA1*, *EFNA3*, *EFNA4*, *EFNB1*, and *EFNB2* are significantly associated with the epithelioid subtype, whereas the spindle subtype demonstrated the lowest expression levels of these genes (Figure 9).

### 2.5. EPH/EFN Expression Patterns in Different Stages of Uveal Melanoma

We used UALCAN to profile EPH expression in different pathological stages of UVM and found that the patients with metastatic disease (Stage 4) had significantly different levels of EPH receptors compared to Stage 3 patients. Specifically, most EPHs (*EPHA3*, *EPHA4*, *EPHA5*, *EPHA8*, *EPHA10*, *EPHB1*, and *EPHB6*) are overexpressed in Stage 4 patients, whereas *EPHA1*, *EPHA2*, *EPHA7*, *EPHB3*, and *EPHB4* expression levels are lower in metastatic patients compared to Stage 3 patients. *EPHB3* expression is associated with advanced stages, whereas the expression of EPHB3 is inversely correlated to a patient’s stage. Similarly to EPH receptors, EFN gene expression (*EFNA1*, *EFNA2*, *EFNA3*, *EFNA4*, *EFNA5*, *EFNB2*, and *EFNB3*) is significantly upregulated in metastatic UVM in comparison with Stage 3 disease, whereas only *EFNB1* expression is downregulated in Stage 4, compared to Stage 3, patients.

Table 1 summarizes the findings of our study and highlights the members of the EPH/EFN family that warrant further investigation in UVM. The chromosomal locations of all *EPH* and *EFN* genes were compiled to facilitate the interpretation of potential associations between CNA and transcriptomic changes.

### 2.6. Evaluation of EPH/EFN Genes’ Correlations with Survival in the TCGA-SKCM (Skin Cutaneous Melanoma) Cohort

**Figure 10:**
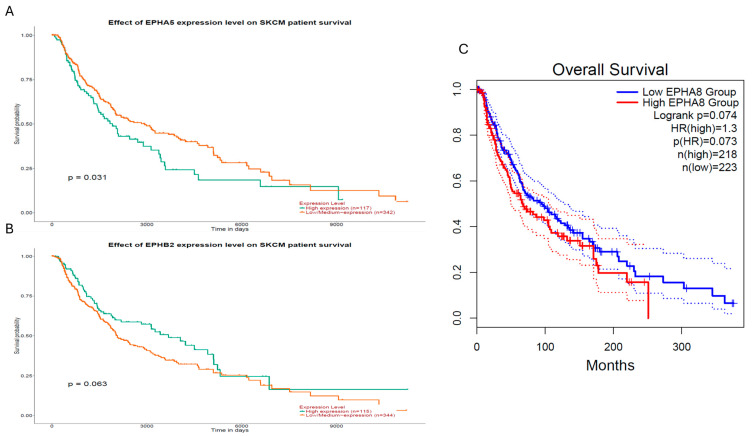
Survival analysis of *EPHA5*, *EPHB2*, and *EPHA8* expression levels in the TCGA-SKCM cohort. Kaplan–Meier survival curves generated using GEPIA2 (**C**) and UALCAN (**A**,**B**), illustrating the prognostic relevance of *EPH/EFN* gene expression levels in cutaneous melanoma (TCGA-SKCM). (**A**) High *EPHA5* expression was significantly associated with reduced overall survival (log-rank *p* = 0.031; n = 117 high, n = 342 low/medium). (**B**) High *EPHB2* expression showed a borderline association with poorer survival (log-rank *p* = 0.063; n = 115 high, n = 344 low/medium). (**C**) High *EPHA8* expression displayed a non-significant trend toward reduced survival (log-rank *p* = 0.074; HR = 1.3; *p*(HR) = 0.073; n = 218 high, n = 223 low). Dotted lines represent 95% Confidence Intervals.

To determine whether the potentially prognostic *EPH/EFN* genes identified in UVM display similar associations with survival in skin cutaneous melanoma (SKCM), we performed a survival analysis with GEPIA2 and UALCAN online tools (Figure 10). Among all *EPH/EFN* members associated with OS or PFI/DFS in UVM, only three genes, *EPHA5*, *EPHB2*, and *EPHA8*, showed association with survival in SKCM (Figure 10). Specifically, high *EPHA5* expression was significantly associated with reduced overall survival (log-rank *p* = 0.031) (Figure 10A). *EPHB2* demonstrated a borderline association (log-rank *p* = 0.063) (Figure 10B), whereas *EPHA8* also exhibited a trend toward poorer prognosis (log-rank *p* = 0.074; HR = 1.3) (Figure 10C). These results indicate that *EPH/EFN* genes may have specific roles in the development and progression of UVM. Consistent with this observation, no statistically significant differences in gene expression were detected across different tumor stages in the SKCM dataset, further suggesting that these *EPH/EFN* alterations do not play a stage-dependent role in cutaneous melanoma.

## 3. Discussion

In this study, we used open-access bioinformatics tools to analyze the TCGA-UVM dataset, the largest available dataset of UVM patients. This is a pilot study on the potential roles of *EPH/EFN* in UVM, and our results suggest that a subset of *EPH/EFN* genes is overexpressed in UVM patients with *BAP1* mutations, Cluster 3 or 4 UVM, and UVM with 8q gain. High expression levels of these *EPH/EFN* genes are associated with worse prognoses. Low expression levels of these *EPH/EFN* genes (for example, in patients with *SF3B1* and *EIF1AX* mutations) are associated with better prognoses. A comparative survival analysis in cutaneous melanoma showed that these gene sets do not have prognostic importance in skin melanoma. We profiled genomic alterations and PVs of *EPH/EFN* genes in UVM patients, and we highlighted the EPHs and EFNs that were correlated with poor survival, aggressive histological subtype, and advanced stage. Genomic alterations of EPHs/EFNs are present in approximately 10% of patients, and the vast majority of them are deep deletions. The only finding that has been classified by OncoKB is the homodeletion of *EPHA7*, considered likely pathogenic, whereas the rest of the reported CNAs are of unknown significance. We found that *EPHA7* expression is not associated with OS, in accordance with the study of Gajdzis et al., who analyzed a cohort of 94 patients with choroidal UVM [43], while, in contrast to the aforementioned study [43], our analysis demonstrated that *EPHA7* expression is associated with reduced PFI. EPHA7 has been found dysregulated in many cancers and may display both a pro-oncogenic and an anti-oncogenic activity [43,54].

We also identified two pathogenic variants, a nonsense mutation in *EPHA4* and a missense mutation in *EFNA4*. Notably, *EPHA4* was also associated with reduced DFS, further supporting its potential role in UVM progression. Furthermore, GEPIA3 and GSCA analyses revealed a significant association of *EPHA4* expression with reduced OS. Our analysis, based on data extracted from the TCGA cohort, demonstrates highest expression of *EPHA4* in Stage 4 patients, and a link between overexpression of *EPHA4* and poor clinical outcome, in accordance with the study of Pergaris et al., in a cohort of 44 UVM patients, which revealed that high expression of EPHA4 is associated with lower OS and metastatic spread [44]. However, the TCGA data highlight that *EPHA4*, together with *EPHA2*, is overexpressed in the epithelioid compared to the spindle and mixed subtypes, in contrast to Pergaris et al. who found no correlation between EPHA2 and EPHA4 expression levels and histological subtype of UVM [44]. The *EPHA4* mutation has also been reported in cutaneous melanomas and characterized as a negative prognostic factor for gastric cancer patients [55], whereas it is associated with better OS in lung cancer patients [56].

*EPHA5* expression correlates with OS/PFI according to our results. More specifically, our analysis on data from the TCGA cohort reveals the highest expression of *EPHA5* in metastatic UVM and suggests an association of *EPHA5* overexpression with shorter OS (GEPIA, GSCA) and DFS/PFI (GEPIA and GSCA). However, a study by Gajdzis et al. showed that high expression of EPHA5 is associated with the absence of metastasis, better OS, and a trend towards prolonged DFS [43]. A potential explanation for this discrepancy is that Gajdzis et al. studied protein expression, whereas we analyzed transcriptomic data. High EPHA5 expression was consistently associated with worse overall survival in UVM patients across GEPIA2 and GSCA datasets. These results are in agreement with previous immunohistochemical evidence by Gajdzis et al. [43], who also reported EPHA5 overexpression in a subset of patients.

Our analysis further indicated that *EPHA8* overexpression is associated with poor survival (OS and DFS), and with the more aggressive epithelioid histological subtype. Our analysis also revealed that several members of gene set 1 and gene set 2 show differential expression across UVM histopathological subtypes. Specifically, EPHA4, *EPHA5*, *EPHA7*, *EPHA8*, *EFNA2*, and *EFNB2* were significantly upregulated in epithelioid and mixed tumors compared with the spindle subtype, as shown in Figure 8 and Figure 9. Given that epithelioid morphology is strongly associated with aggressive behavior and poor prognosis, these findings support a potential link between the prognostic gene sets and more aggressive histopathological features. EPHA8 is considered a negative prognostic factor for ovarian cancer patients [57].

Exactly like the aforementioned *EPHA8*, *EFNB2* overexpression is also associated with poor survival (OS and DFS), and with the more aggressive epithelioid histological subtype. EFNB2 overexpression is associated with poor outcome in thyroid cancer [58], glioblastoma [59], and cholangiocarcinoma [60], as well as pancreatic [61], bladder [62], ovarian [63], and endometrial carcinomas [64], whereas it is associated with a favorable prognosis in breast cancer [65]. Recently, Gentien et al. revealed that *EPHA8* and *EPHA4* expression levels were consistently upregulated in single UVM cells isolated from xenograft models derived from patients with epithelioid, metastatic UVM compared to normal uveal melanocytes [35].

All of the aforementioned genes, namely *EPHA8*, *EFNB2*, *EPHA5*, *EPHB2,* and *EPHA4* (excepting *EPHA7*) are significantly overexpressed in metastatic disease in comparison with advanced non-metastatic disease.

Our study examines, for the first time, the potential association of the whole EPH/EFN family with clinicopathological parameters (histology and survival) of UVM. However, it is a descriptive bioinformatic analysis based on data mining from the TCGA-UVM dataset with open-access web tools, with several limitations. We did not include additional bioinformatics pipeline, and we did not perform multivariate Cox regression or other analyses that would verify the prognostic capacity of the EPH/EFN family members in UVM. Therefore, we highlight genes of interest, without being able to confirm their prognostic importance. The gene sets that are associated with reduced survival do not represent a signature with a confirmed risk score. Univariate, multivariate, and receiver operating characteristic (ROC) analyses in a testing/validation cohort are required to confirm the prognostic and predictive significance of the genes highlighted by our study. A differential GSEA (Gene Set Enrichment Analysis) of the UVM tumors that express high and low levels of the key EPH/EFN genes would enable us to better understand the pathogenesis and progression of UVM. Another limitation of our work is that it is restricted to data mining from one dataset (the TCGA-UVM dataset),and does not include experimental (wet lab) data that verify the *in-silico* findings. The methodology of our analysis is based on transcriptomic data, whereas the analysis of protein expression levels would be more appropriate for the evaluation of the EPH/EFN family role in UVM. Additionally, we used different platforms with heterogeneous methods for data mining, introducing inconsistencies in some analyses.

Although the roles of EPH/EFN family members in cancer progression remain to be elucidated, there is accumulating evidence that the dysregulation of these axon guidance pathways significantly impacts the OS of cancer patients [66]. The pool of our analyses indicates members of the EPH/EFN family that need to be investigated further, in larger cohorts of UVM patients.

## 4. Materials and Methods

Our results are largely based upon data generated by the TCGA research network https://www.cancer.gov/ccg/research/genome-sequencing/tcga (last accessed on 18 December 2025). We used several online platforms to explore and analyze the TCGA-UVM dataset.

### 4.1. cBioPortal Analysis

The cBioPortal for Cancer Genomics (http://cbioportal.org, last accessed on 18 December 2025) is an open-access online platform for the analysis of cancer genomic data from TCGA projects and other studies [67]. We used cBioPortal to profile the pathogenic variants (PVs) and copy number alterations (CNAs) of EFN and EPH genes in UVM. Specifically, we included, in our analysis, two datasets termed “TCGA PanCancer Atlas (80 cases)” [68] and “QIMR Oncotarget (28 cases)” [69], and we omitted the TCGA-Firehose analysis study because it was a provisional analysis that was refined in the TCGA-PanCancer Atlas analysis of the same cohort of patients. We selected OncoPrint mode for data presentation and alterations–mutations were annotated by OncoKB [70]. OncoKB (https://www.oncokb.org/, last accessed on 18 December 2025) is a precision oncology knowledge base that provides curated information on the oncogenic effects and clinical significance of somatic genomic alterations. Within cBioPortal, OncoKB annotates mutations and copy number alterations as oncogenic, likely oncogenic, or of unknown significance based on existing biological and clinical evidence. In our analysis, all detected PVs and CNAs were interpreted using OncoKB classifications. Moreover, PVs present in UVM patients were cross-checked with the Catalogue of Somatic Mutations In Cancer (COSMIC) database v98 [71]. Chromosomal locations of all *EPH* and *EFN* genes were retrieved from the Ensembl Genome Browser (GRCh38/hg38), available at: https://www.ensembl.org/Homo_sapiens/Info/Index, last accessed on 18 December 2025).

### 4.2. GEPIA2 and GEPIA3 Analysis

GEPIA2 (Gene Expression Profiling Interactive Analysis 2) (http://gepia2.cancer-pku.cn/#index) (last accessed on 18 December 2025) and GEPIA3 (https://gepia3.bioinfoliu.com/) (last accessed on 18 December 2025) are online servers for customized gene expression and survival analysis of TCGA data [49]. We used GEPIA2 and GEPIA3 to profile the prognostic value of EPH and EFN expression levels in UVM. We analyzed the OS and the DFS of UVM patients with high and low expression levels of these genes. Kaplan–Meier curves were generated, using a median group cut-off to stratify patients (50% of patients with high and 50% of patients with low expression levels). GEPIA3 was used to plot the expression levels of gene sets 1 and 2 in UVM patients with different mutations. *p*-values and Hazard Ratios (HRs) were calculated by GEPIA2 and GEPIA3 using log-rank tests and Cox regression models. *p* < 0.05 was considered statistically significant.

### 4.3. UALCAN Analysis

UALCAN (the **U**niversity of **AL**abama at Birmingham **CAN**cer data analysis portal) (https://ualcan.path.uab.edu/) (last accessed on 18 December 2025). is a web platform for mining and analysis of TCGA datasets [72]. We used UALCAN to analyze the gene expression levels of EFN ligands and EPHs in different stages and histological subtypes of UVM. Data were depicted as boxplots, and statistical analysis was performed by UALCAN. Student’s *t*-test was employed to compare between groups, and *p* < 0.05 was considered as statistically significant.

### 4.4. GSCA Analysis

GSCA (Gene Set Cancer Analysis) is an online platform (https://guolab.wchscu.cn/GSCA/#/) (last accessed on 18 December 2025) that integrates genomic (including pharmacogenomic and immunogenomic), transcriptomic, and clinicopathological data from TCGA and other online databases [73,74]. We used GSCA to perform single-gene expression and survival analysis to test if we could confirm results generated by GEPIA. Kaplan–Meier curves were generated, using a median group cut-off, and the calculations of *p*-values and HRs were based on log-rank tests and Cox regression models. Moreover, we performed a GSCA analysis of gene sets, which included members of the EPH/EFN family that were significantly associated with OS or DFS in all platforms. This GSCA analysis was based on the Gene Set Variation Analysis (GSVA) method, a well-established method that calculates the integrated expression levels of all the genes that constitute the set [75]. Samples were divided into high (50% of the cohort) and low (50% of the cohort) GSCA score groups, and survival analysis (overall and progression-free survival—PFS) was performed. Log-rank *p* values and HRs were calculated by the software. *p*-values < 0.05 were considered as statistically significant.

### 4.5. TCGEx Analysis

TCGEx is a very recent online platform for analysis of cancer transcriptomic data (https://tcgex.iyte.edu.tr/) (last accessed on 18 December 2025). We used TCGEx to perform a ROC analysis of gene sets 1 and 2 in TCGA-UVM, and also to perform a correlation plot analysis (Spearman correlation) of the EPHs/EFNs of interest, with different molecular clusters of UVM and CNAs of 8q chromosome [51].

### 4.6. ShinyGeo Analysis [52]

ShinyGeo (https://gdancik.shinyapps.io/shinyGEO/) (accessed on 18 December 2025) is an online tool that enables the analysis of publicly deposited gene expression datasets (GEO, Gene Expression Omnibus) from TCGA and other cancer studies. We used ShinyGeo to download and analyze UVM dataset GSE84976 (https://www.ncbi.nlm.nih.gov/geo/query/acc.cgi?acc=GSE84976) (accessed on 18 December 2025).

## 5. Conclusions

We used online open-access bioinformatics tools to analyze publicly deposited genomic and transcriptomic data from the TCGA-UVM cohort. Our findings suggest that a subset of EPH and EFN ligands may be associated with worse clinical outcomes (EPHA4, EPHA5, EPHA7, EPHA8, EPHB2, EFNA2, and EFNB2), and an aggressive histological subtype (EPHA2, EPHA4, EPHA8, EPHB4, EFNA1, EFNA3, EFNA4, and EFNB2). Additional bioinformatic and experimental studies are required to validate these findings and evaluate the utility of EPHs/EFNs as potential biomarkers for UVM.

## Figures and Tables

**Table 1:** Genomic alterations and clinical associations of *EPH/EFN* genes in UVM. Summary of pathogenic variants (PVs), copy number alterations (CNAs), and their associations with overall survival (OS), disease-free survival/progression-free interval (DFS/PFI), and histological subtype in TCGA-UVM. Downward arrows (↓) indicate reduced survival. The asterisk indicates nonsense mutation.

Gene (Location)	Genomic Alteration (Percentage of Patients)	OS	DFS/PFI	UVM Histology
EPHA1 (7q34–q35)	Homodeletion (0.9%)			
EPHA2 (1p36.13)				Epithelioid
EPHA4 (2q36.1)	Homodeletion (0.9%), p.R230* nonsense PV (c.688C>T) (0.9%)	↓	↓	Epithelioid
EPHA5 (4q13.1–q13.2)		↓	↓	
EPHA7 (6q16.1)	Homodeletion (4%)		↓	
EPHA8 (1p36.12)		↓	↓	Epithelioid
EPHB2 (1p36.13)	Homodeletion (1.9%)	↓	↓	
EPHB4 (7q22.1)				Epithelioid
EPHB6 (7q34)	Homodeletion (0.9%)			
EFNA1 (1q22)				Epithelioid
EFNA2 (19p13.3)	Amplification (0.9%)		↓	
EFNA3 (1q21.3)				Epithelioid
EFNA4 (1q21.3)	R196H missense PV (587G>A) (0.9%)			Epithelioid
EFNB2 (13q33.3)		↓	↓	Epithelioid

## Data Availability

Extracted/generated/analyzed datasets that are included in this study are available in the GEPIA2 (http://gepia2.cancer-pku.cn/#index) (accessed on 18 December 2025), GEPIA3 (https://gepia3.bioinfoliu.com/) (accessed on 25 December 2025), UALCAN (https://ualcan.path.uab.edu) (accessed on 18 December 2025) (cBioPortal (https://www.cbioportal.org) (accessed on 18 December 2025), GSCA (https://guolab.wchscu.cn/GSCA/#/) ((accessed on 18 December 2025)) and TCGEx (https://tcgex.iyte.edu.tr/) (accessed on 18 December 2025). online repositories.

## Supplementary Materials

The following supporting information can be downloaded at: https://www.mdpi.com/article/10.3390/ijms27010442/s1.
