# Supplementary material for: Ephrin Receptors and Ephrin Ligands in Uveal Melanoma: A Big Data Analysis Using Web Resources
Source: Int J Mol Sci. 2025 Dec 31;27(1):442. doi: 10.3390/ijms27010442 (PMC12786134; doi:10.3390/ijms27010442)
Supplement: Supplementary file 1 [file ijms-27-00442-s001.zip › ijms-3974464-supplementary.pdf]

Figure S1

A

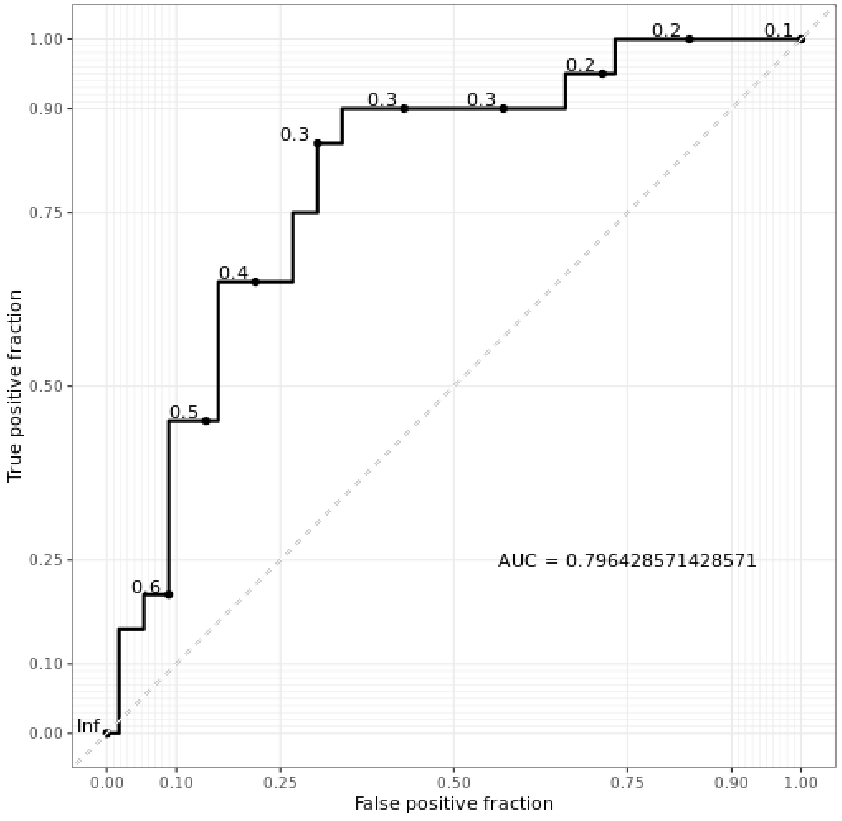

AUC = 0.796428571428571
True positive fraction
False positive fraction
0.00
0.10
0.25
0.50
0.75
0.90
1.00
Inf
0.6
0.5
0.4
0.3
0.3
0.3
0.2
0.2
0.1

B

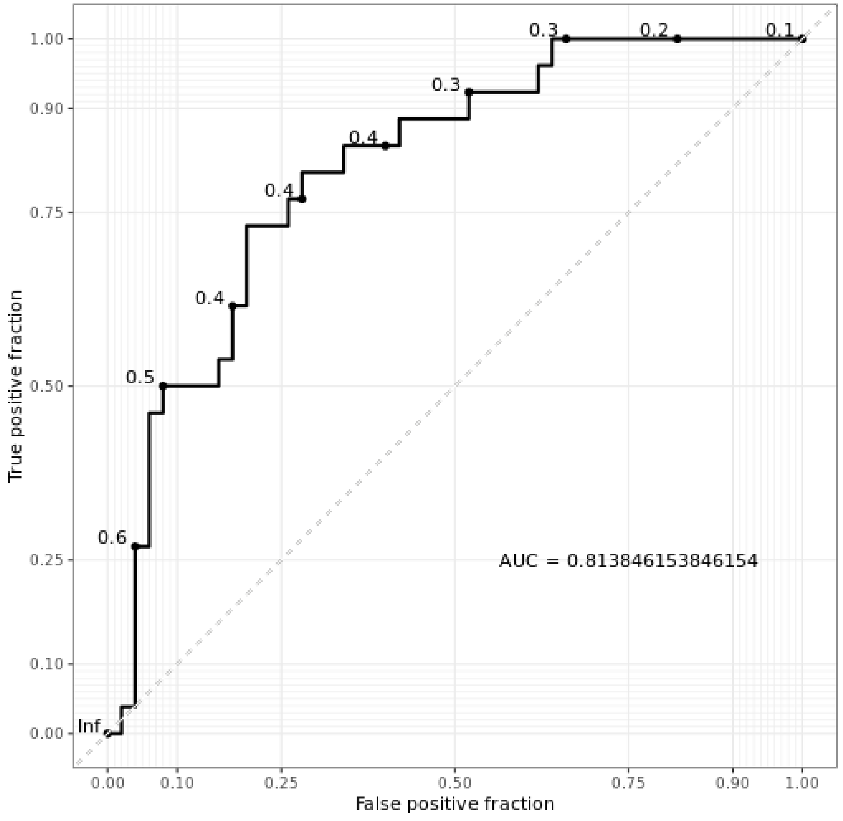

AUC = 0.813846153846154
True positive fraction
False positive fraction
0.00
0.10
0.25
0.50
0.75
0.90
1.00
Inf
0.6
0.5
0.4
0.4
0.4
0.3
0.3
0.2
0.1

Figure S2

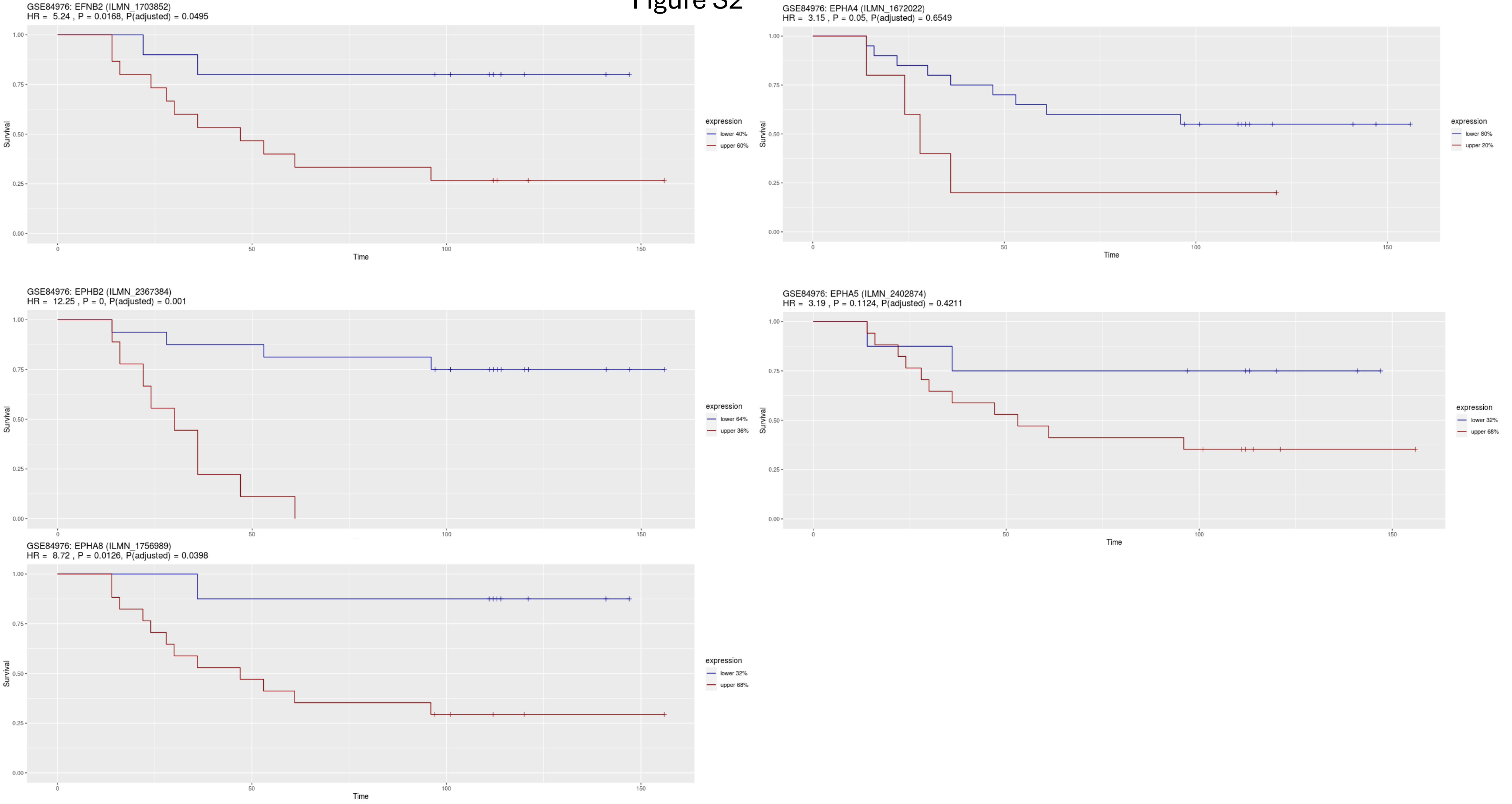
GSE84976: EFNB2 (ILMN_1703852)
HR = 5.24 , P = 0.0168, P(adjusted) = 0.0495
Survival
Time
expression
lower 40%
upper 60%
GSE84976: EPHA4 (ILMN_1672022)
HR = 3.15 , P = 0.05, P(adjusted) = 0.6549
Survival
Time
expression
lower 80%
upper 20%
GSE84976: EPHB2 (ILMN_2367384)
HR = 12.25 , P = 0, P(adjusted) = 0.001
Survival
expression
lower 64%
upper 36%
GSE84976: EPHA5 (ILMN_2402874)
HR = 3.19 , P = 0.1124, P(adjusted) = 0.4211
Survival
Time
expression
lower 32%
upper 68%
GSE84976: EPHA8 (ILMN_1756989)
HR = 8.72 , P = 0.0126, P(adjusted) = 0.0398
Survival
Time
expression
lower 32%
upper 68%

Table S1

Gene expression significantly associated with OS

genename	p-value	q-value
EPHA4	0.00329	0.0145
EPHA5	0.00689	0.0232
EPHA8	0.0114	0.0323
EPHB2	9.33E-05	0.00154
EFNB2	0.00457	0.0178

Gene expression significantly associated with PFI

genename	p-value	q-value
EPHA4	0.000588	0.00404
EPHA5	0.00238	0.00921
EPHA7	0.0168	0.0323
EPHA8	0.00065	0.00423
EPHB2	0.00235	0.00915
EFNA2	0.00961	0.0226
EFNB2	0.00123	0.00615
